# Identification of* Fasciola* Species Isolates from Nghe An Province, Vietnam, Based on ITS1 Sequence of Ribosomal DNA Using a Simple PCR-RFLP Method

**DOI:** 10.1155/2018/2958026

**Published:** 2018-12-04

**Authors:** Do Ngoc Anh, Le Tran Anh, Le Quoc Tuan, Nguyen Duy Bac, Tran Viet Tien, Vu Thi Binh Phuong, Tran Thanh Duong, Nguyen Khac Luc, Nguyen Ba Quang

**Affiliations:** ^1^Department of Medical Parasitology, Vietnam Military Medical University, Hanoi 100000, Vietnam; ^2^Department of Anatomy, Vietnam Military Medical University, Hanoi 100000, Vietnam; ^3^Department of Infectious Diseases, Vietnam Military Medical University, Hanoi 100000, Vietnam; ^4^Department of Medical Parasitology, Thai Binh University of Medicine and Pharmacy, Thai Binh 410000, Vietnam; ^5^National Institute of Malariology Parasitology and Entomology Vietnam, Hanoi 100000, Vietnam; ^6^Department of Laboratory Medicine, Vietnam National Hospital of Acupuncture, Hanoi 100000, Vietnam

## Abstract

Fascioliasis—a disease caused by* Fasciola *spp. (Platyhelminthes: Trematoda: Digenea)—is considered as the most important helminthic infection of bovine, sheep, and buffalo in Vietnam. The aim of this study is to detect the genotype of* Fasciola *spp. isolated from bovine and buffalo in the Nghe An province, central Vietnam, using PCR-RFLP and sequence analysis of the first nuclear ribosomal internal transcribed spacer (ITS1). Adult* Fasciola *spp. were isolated from bile ducts of bovine and buffalo in Nghe An province, Vietnam. Overall, 96 adult flukes from livers of slaughtered animals were collected from abattoirs of different areas. They included 7 samples from infected bovine and 89 samples from infected buffalo. 96/96 samples were identified as* Fasciola* species by ITS1 of rDNA. In this study, a PCR-RFLP method was used to distinguish between* F. hepatica *and* F. gigantica* in ITS1 of rDNA (680 bp) with* Rsa*I restriction enzyme. RFLP pattern with* Rsa*I produced a consistent pattern of 360, 100, and 60 bp fragments in* F. hepatica*, whereas* F. gigantica* worms had a profile of 360, 170, and 60 bp in size, respectively. The results showed that using PCR-RFLP based on the first internal transcribed spacers (ITS1) of the ribosomal RNA revealed that 93 out of 96 isolates were of* Fasciola gigantica* type, whereas three isolates presented an intermediate* Fasciola*. In the present study,* F. gigantica* and intermediate form were coexisting in bovine and buffalo in the Nghe An province of central Vietnam, whereas* F. hepatica *was not detected.

## 1. Introduction

Fascioliasis, a disease caused by the liver flukes of the genus *Fasciola,* is one of the most important food- and water-borne parasitic zoonoses.* F. hepatica* and* F. gigantica* are two main species which infect humans and animals.* F. hepatica* has a worldwide distribution and both species exist in the tropical and subtropical regions of Africa and Asia [1, 2]. Differentiation of these two species, based on morphological characteristics such as the ratio of body length to width, is difficult due to the variation in their size, particularly with respect to the age of flukes, involved host species, and fixation techniques used [3]. Because of the limitations of morphological methods, several molecular approaches, using different molecular targets, have been developed for the differentiation of* F. hepatica *and* F. gigantica *[4].

Molecular approaches can be properly distinguished by DNA sequencing of first internal transcribed spacers (ITS1), ITS2, and 28S ribosomal ribonucleic acid genes [5–7]. Several studies using ITS1 of rDNA showed that* F. hepatica*,* F. gigantica*, and their intermediate forms exist in different countries including Vietnam [8–10]. Nevertheless, no reports in bovine and buffalo from Nghe An province exist. This province is located in the centre of Vietnam. Previously, the Nghe An province has been known as an important endemic area for fasciolosis in bovine [11], but very limited data is available on molecular characterization of* Fasciola* spp. of buffalo in this province.

Therefore, this study has been performed to detect the genotype of* Fasciola *spp. isolated from bovine and buffaloes in the Nghe An province, central Vietnam, using PCR-RFLP and sequence analysis of the first nuclear ribosomal internal transcribed spacer (ITS1).

## 2. Materials and Methods

### 2.1. Flukes

**Figure 1:**
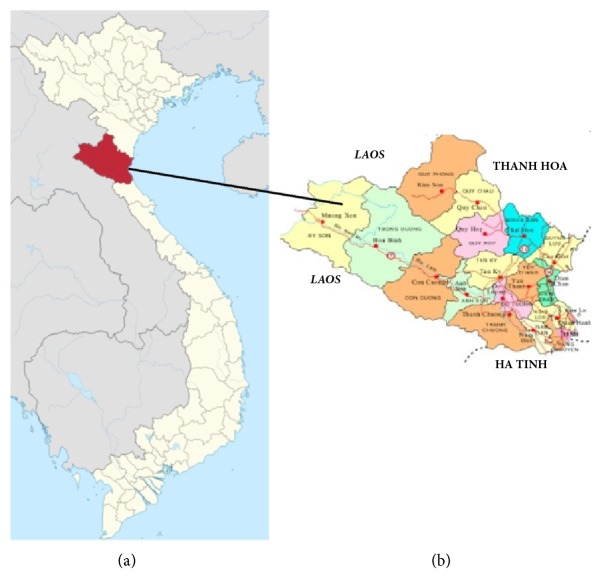
Map of Vietnam (a). Nghe An province, central Vietnam (b).

Adult flukes (89 flukes from 5 buffalo and 7 flukes from one bovine) were isolated from bile ducts at slaughterhouses from the Nghe An province in Vietnam (Figure 1), where human cases of fasciolosis have been recently reported. Flukes were washed extensively in physiological saline and subsequently fixed in 70% ethanol and preserved at 4°C until extraction of genomic DNA.

### 2.2. DNA Extraction

Approximately 25mg tissue samples were removed from each adult fluke. Total DNA was extracted using QIAamp DNA Mini Kit (No. 51304, Qiagen, Germany) according to the manufacturer's instructions. Extracted DNA was diluted in double distilled water and maintained at -20°C until used in the PCR.

### 2.3. PCR Amplification

To amplify an approximate 680 bp region of the ITS1 sequence, PCR was performed using a set of ITS1-F (5'-TTG CGC TGA TTA CGT CCC TG-3) and ITS2-R (5'-TTG GCT GCG CTC TTC ATC GAC-3') (Integrated DNA Technologies, USA) as sense and antisense primers (Itagaki et al., 2005) [12], respectively. Total volume of PCR reaction was 50 *μ*l containing 5 *μ*l of DNA solution, 25 *μ*l mastermix 2X (Thermo Fisher Scientific, USA), 1.0 *μ*l of each primer (0.2 *μ*M), and 18.0 *μ*l of distilled water. PCR amplification was performed in Thermo Mastercycler Gradient (Thermo Fisher Scientific, USA). The reaction cycle was as follows: an initial denaturation step at 94°C for 5 minutes, followed by 35 cycles of 94°C for 30 seconds (denaturation), 55°C for 30 seconds (annealing), and 72°C for 60 seconds (extension) and a final extension of 72°C for 15 minutes.

### 2.4. Restriction Fragment Length Polymorphism (RFLP) Analysis

RFLP was performed according to the method described by Ichikawa M et al. to distinguish* F. hepatica *from* F. gigantica* in ITS1 with *Rsa*I enzyme [8]. To perform RFLP assay, total volume of 16 *μ*l, including 5 *μ*l of ITS1 PCR product, was added with 1 *μ*l of* Rsa*I, 1 *μ*l of 10X Tango buffer (Thermo Fisher Scientific, USA), and 9 *μ*l of distilled water. The tubes were incubated at 37°C for 12 hours to ensure full cutting of fragments, and* Rsa*I was heat-inactivated at 65°C for 15 min. 6 *μ*l of each product and 1 *μ*l of loading dye buffer were electrophoresed on 2% agarose gel in TBE buffer at 100 V for 60 min and visualized by UV illumination (UVP, Canada) after ethidium bromide staining. The size of each band was determined by a 50 bp plus ladder molecular weight marker (Thermo Fisher Scientific, USA). DNA types of* Fasciola *spp. were distinguished according to fragment patterns, three bands of 360, 100, and 60 bp fragments in* F. hepatica*, whereas* F. gigantica* worms had a profile of 360, 170, and 60 bp in size, respectively.

### 2.5. DNA Sequencing

PCR products of ITS1 from two isolates, 10B-NA1.2 isolated from bovine and 15Tr-NA5 isolated from buffalo, were sent to First BASE Laboratories Sdn Bhd-service (Kembangan 43300, Selangor, Malaysia) for purification and automatic sequencing in both directions, using the same primers which were used in the PCR. Sequences were read on ABI 3130 Genetic Analyzer software (SeqScape 2.1). The accuracy of data was confirmed by two-directional sequencing. Representative sequences were deposited in the GenBank under accession numbers MH790325 and MH790326.

### 2.6. Sequence and Phylogenetic Analyses

The obtained sequences were analyzed independently by being compared with related sequences available in the GenBank database using BLAST guidelines (http://blast.ncbi.nlm.nih.gov/Blast.cgi). Neighbor joining (NJ) and maximum likelihood (ML) based on Tamura-Nei model phylogenetic tree of ITS1 sequences were constructed using Mega version 7.09 software. Bootstrap analyses (1,000 replications) were carried out to determine the robustness of the finding. rDNA sequences of* Paragonimus westermani *(AF040935.1) were used as an outgroup.

## 3. Results

### 3.1. PCR and PCR-RFLP

**Figure 2:**
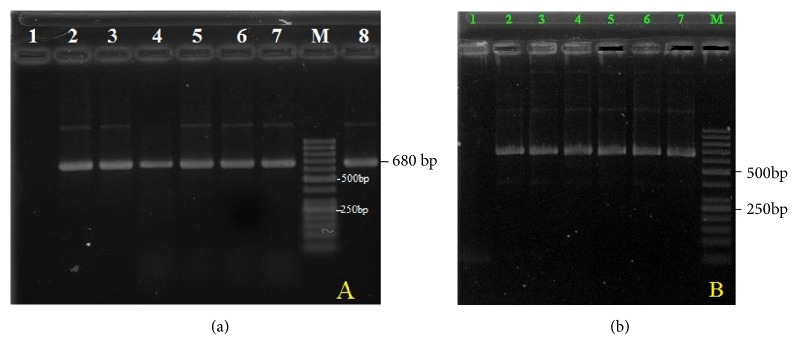
PCR patterns of amplified ITS1 ribosomal region. Lane M: 50 bp ladder molecular weight marker; lane 1 (a, b): negative control; lanes 2-8 (a) and 2-7 (b) denoted to different fluke samples amplified as a single band of 680 bp of bovine and buffalo in Nghe An province.

**Figure 3:**
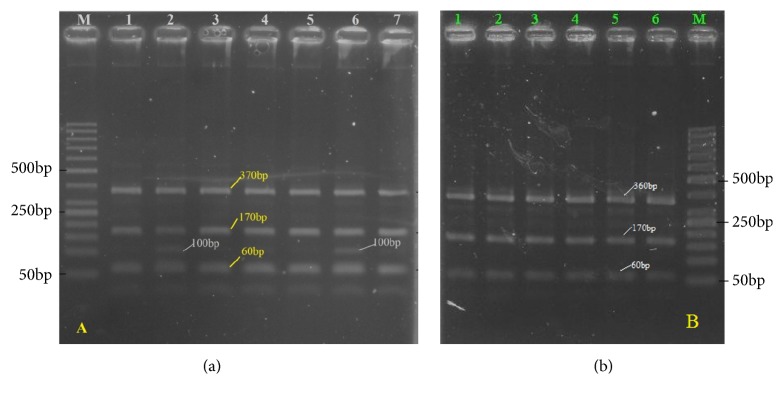
RFLP pattern of PCR products of liver flukes collected from bovine (a) and buffalo (b) in Nghe An province, Vietnam, after digestion with* Rsa*I enzyme. Lane M: 50 bp ladder molecular weight marker; lanes 1, 3, 4, 5, and 7 (a) denoted to those of* F. gigantica*; lanes 2 and 6 (b) denoted to those of* Fasciola *sp. (intermediate form); lanes 1-6 (b) denoted to those of* F. gigantica.*

A region of approximately 680bp of the ITS1 of rDNA in 96 samples was successfully amplified (Figure 2) as predicted. Negative control did not produce any band on the gels. PCR-RFLP bands profile of* Fasciola *with restriction enzymes* Rsa*I was performed. The results of PCR products digestion with* Rsa*I were approximately 60, 100bp, and 360bp fragments for* F. hepatica*; 60, 170 bp, and 360 bp for* F. gigantica*; and 60, 100, 170, and 370 bp for intermediate form (Figure 3).

Table 1 illustrates the identified liver flukes in relation to their origins and their definitive hosts. Accordingly, 96* Fasciola* isolates were studied from bovine and buffalo of Nghe An province, Vietnam. Out of 96 analyzed specimens from Nghe An province, 93 (96.88%) flukes were identified as* F. gigantica* and 3 (3.12%) as intermediate* Fasciola* (Table 1).

### 3.2. Complete ITS1 Sequencing of rDNA

DNA sequencing of two ITS1 was complete. BLAST results showed that* F. gigantica *from Thailand (AB207144.1) possessed the sequence most similar to those in these 2 worms with 99.31% identity for FasVN09 (10B-NA1.2) isolate (MH790325) and 100% identity for FasVN14 (15Tr-NA5) isolate (MH790326).

**Figure 4:**
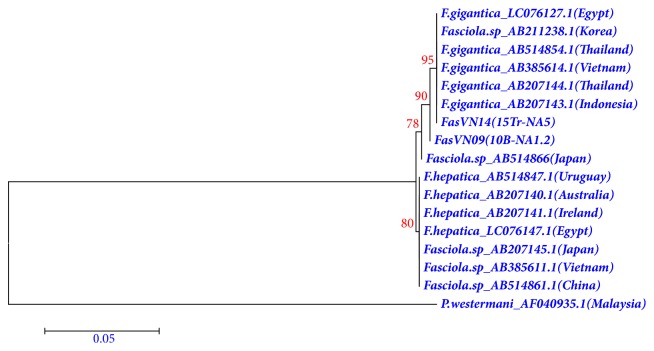
Phylogenetic relationship of ITS1 sequences of* Fasciola* from Vietnam using Neighbor-Joining Tree method.* Paragonimus westermani *(AF040935.1) was used as the outgroup.

Phylogenic tree constructed by using ITS1 sequences of the* Fasciola* species is shown in Figure 4. All the isolates of* Fasciola* in different countries clustered together, supported by high bootstrap value (>78%). The phylogenetic tree constructed by Neighbor-Joining Tree method supports the blast results by showing similarity between ITS1 sequences (Nghe An province, Vietnam) and ITS1 in Egypt (LC076127.1), Thailand (AB514854.1), Vietnam (AB385614.1), and Indonesia (AB207143.1). The nucleotide sequence data obtained in current study have been deposited in the GenBank under accession numbers MH790326.1 (15Tr-NA5 isolate) and MH790325.1 (10B-NA1.2 isolate).

## 4. Discussion

DNA-based molecular methods are accurate and reliable for understanding the different species of* Fasciola* and considered control procedures in endemic areas [13, 14]. PCR-RFLP assay is a powerful method to distinguish between* F. hepatica* and* F. gigantica*. In this study, PCR-RFLP based on the partial rDNA of ITS1 and restriction* Rsa*I enzyme was used for differentiation and identification of* Fasciola* species in Nghe An province. These techniques have been utilized for differentiation among* Fasciola* species based on the profiles generated by the effects of endonucleases on ITS genes of these parasites [15]. Ichikawa et al. (2011) used* Rsa*I enzyme based on ITS1 region to specifically distinguish* F. hepatica* and* F. gigantica *in Myanmar and did not report* F. hepatica* in their study. In a study in Iran, Aryaeipour et al. (2014) showed that* Rsa*I restriction enzyme may be utilized for the differentiation of two species [16]. Saki et al.* (2011)* used* Ava*II and* Dra*II to differentiate between* F. hepatica* and* F. gigantica* in 28S DNA [17]. Ghavami et al. (2009) showed that a digested pattern of 230, 340, and 341bp is specific to* F. hepatica* species and has no effect on* F. gigantica *[18]. Ashrafi et al. (2004) used ITS2 nucleotide sequencing to identify* Fasciola *spp. In their study, two species of* F. hepatica* and* F. gigantica* were seen in Guilan, northern Iran [19].

Our study did detect* F. gigantica *and mixed patterns in Nghe An province but not* F. hepatica*. The results of our study were similar to those of some previous researches in Vietnam [20, 21]. The majority of* Fasciola*species in this study were molecularly identified as* F. gigantica *which was concordant with the knowledge that* F. gigantica* mainly infected buffalo and bovine in Vietnam. In addition the hybrid form of* Fasciola* had been confirmed based on analysis of ITS1 sequence. The intermediate form from a human case and animals had been reported in Vietnam [10, 22]. The absence of* F. hepatica* in the current study was supported by recent studies of a large number of* Fasciola* from animals in the country [10] and some neighboring countries such as Myanmar and Thailand [3, 23]. Intermediate* Fasciola *form has been reported to coexist in areas sympatric for both* F. hepatica* and* F. gigantica* such as some Asian countries (Japan, Korea, China, and Iran) [12]. In this study, the coexistence of intermediate* Fasciola *and* F. gigantica* in the absence of* F. hepatica* was detected in Vietnam as well as in Thailand and Myanmar [3, 23].

## 5. Conclusion

Using ITS1 marker has identified both* F. gigantica* and intermediate form of* Fasciola *coexisting in the Nghe An province of central Vietnam.

## Figures and Tables

**Table 1:** Frequency of *F. gigantica* and *F. hepatica *identified by PCR-RFLP in different animal hosts in Vietnam.

Host	Bovine	Buffalo	Total number of adult flukes
*F. gigantica*	5	88	93
*Fasciola *sp.	2	1	3
*F. hepatica*	0	0	0

Total number	7	89	96

## Data Availability

The data used to support the findings of this study are available from the corresponding author upon request.
